# Prognostic value of pretreatment serum lactate dehydrogenase level in patients with solid tumors: a systematic review and meta-analysis

**DOI:** 10.1038/srep09800

**Published:** 2015-04-22

**Authors:** Jiao Zhang, Yan-Hong Yao, Bao-Guo Li, Qing Yang, Peng-Yu Zhang, Hai-Tao Wang

**Affiliations:** 1 Department of Interventional Oncology, Tianjin Medical University Cancer Institute and Hospital, National Clinical Research Center for Cancer, Tianjin, China; 2 Research Group of Evidence-based Clinical Oncology, Tianjin, China; 3 Tianjin Key Laboratory of Cancer Prevention and Therapy, Tianjin, China; 4 Department of Urologic Oncology, Tianjin Medical University Cancer Institute and Hospital, Tianjin, China; 5 Department of Laboratory Medicine, Tianjin Medical University Cancer Institute and Hospital, Tianjin, China

## Abstract

Although most studies have reported that high serum lactate dehydrogenase (LDH) levels are associated with poor prognosis in several malignancies, the consistency and magnitude of the impact of LDH are unclear. We conducted the first comprehensive meta-analysis of the prognostic relevance of LDH in solid tumors. Overall survival (OS) was the primary outcome; progression-free survival (PFS) and disease-free survival (DFS) were secondary outcomes. We identified a total of 68 eligible studies that included 31,857 patients. High LDH was associated with a HR for OS of 1.48 (95% CI = 1.43 to 1.53; P < 0.00001; I^2^ = 93%), an effect observed in all disease subgroups, sites, stages and cutoff of LDH. HRs for PFS and DFS were 1.70 (95% CI = 1.44 to 2.01; P < 0.00001; I^2^ = 13%) and 1.86(95% CI = 1.15 to 3.01; P = 0.01; I^2^ = 88%), respectively. Analysis of LDH as a continuous variable showed poorer OS with increasing LDH (HR 2.11; 95% CI = 1.35 to 3.28). Sensitivity analyses showed there was no association between LDH cutoff and reported HR for OS. High LDH is associated with an adverse prognosis in many solid tumors and its additional prognostic and predictive value for clinical decision-making warrants further investigation.

Cancer is the leading cause of death in economically developed countries and the second leading cause of death in developing countries^1^. In the United States, a total of 1,660,290 new cancer cases and 580,350 cancer deaths were projected to occur in 2013^2^. In Europe, there were an estimated 3.45 million new cases of cancer (excluding non-melanoma skin cancer) and 1.75 million deaths from cancer in 2012^3^. Furthermore, the global burden of cancer continues to increase, largely because of population growth and increased life-expectancy^3^. Invasion and metastasis are two important hallmarks of cancer and are responsible for the majority of cancer deaths^4^. Although much effort has been devoted to the diagnosis and therapy of cancers, the overall prognosis is still unsatisfactory. A lack of knowledge of molecular biomarkers in cancer has limited the development of personalized therapies and improvements in survival. Therefore, there is an urgent need for universal, effective, readily available and inexpensive biomarkers in solid tumors to identify patients with a poor prognosis so that novel treatments can be initiated earlier.

The metabolism of cancer cells differs from that of normal cells. This is largely because cancer cells exhibit metabolic alterations that are frequently associated with reprogramming. Unlike normal cells, cancer cells preferentially metabolize glucose by glycolysis to generate sufficient energy for the demands of rapid proliferation, even in the presence of adequate oxygen^5^.This phenomenon is known as the Warburg effect and is one of the predominant metabolicalterations that occur during malignant transformation. In this process, transcriptional programs regulated by oncogenes stabilize hypoxia-inducible factor 1 alpha (HIF-1α). HIF-1α contributes to the upregulation of most enzymes involved in the glycolytic pathway, including lactate dehydrogenase (LDH).In the final step of aerobic glycolysis, LDH converts pyruvate tolactate, which is coupled with the oxidation of NADH to NAD+. These metabolic changes are reflected by an elevated serum LDH level^6^(hereinafter LDH).

Elevated LDH has been recognized as a poor prognostic indicator in cancer for many years^7^,^8^,^9^,^10^. LDH has also been incorporated in prognostic scores for several types of cancer^11^. However, the consistency and magnitude of the prognostic impact of LDH are unclear^12^,^13^,^14^. The aim of this study was to review published studies and use standard meta-analytic techniques to quantify the prognostic value of LDH in various solid tumors.

## Methods

### Data sources and searches

This analysis was conducted in line with the Preferred Reporting Items for Systematic Reviews and Meta-Analyses guidelines^15^. PubMed was searched for studies evaluating the LDH and survival in solid tumors from 1978 to 2014. We used various medical subject heading terms, including “l-lactate dehydrogenase”, “prognosis”, “multivariate analysis” and “proportional hazard model”. Title/abstract words included “lactate dehydrogenase”, “LDH”, “prognosis”, “prognose”, “prognostic”, “multivariate analysis”, “proportional hazard model”, “COX proportional hazard model” and “COX models”. The full search strategy is described in the Supplementary Methods (available online).

### Study selection

Inclusion criteria for the primary analysis were as follows: 1) studies of people with solid tumors reporting on the prognostic impact of LDH; 2) prospective or retrospective cohort design with a clearly defined source population and justifications for all excluded eligible cases; 3) sample size greater than 200; 4)statistical analysis using multivariate proportional hazards modeling that adjusted for clinical prognostic factors; and 5) reporting of the resultant adjusted hazard ratios (HRs) and their 95% confidence intervals (CIs) or a P value for overall survival (OS). For the secondary analyses, studies providing a HR for cancer-specific survival (CSS), progression-free survival (PFS), disease-free survival (DFS), or recurrence-free survival (RFS) were included as well.

### Data extraction

OS was the primary outcome of interest. CSS, PFS, and DFS were secondary outcomes. Two authors (J.Z. and H.W.) independently extracted information using predefined data abstraction forms. The following details were extracted: name of first author, year of publication, number of patients included in analysis, disease site, disease stage (non-metastatic, metastatic, mixed [both non-metastatic and metastatic]), study type (prospective or retrospective), cutoff defining high LDH, and HRs and associated 95% confidence intervals for OS, PFS, DFS, or RFS as applicable. HRs were extracted preferentially from multivariate analyses where available. Where several HR values were given in an article, the value adjusted for most confounders was used.

### Data synthesis

The meta-analysis was conducted initially for all included studies for each of the endpoints of interest. Subgroup analyses were conducted for predefined parameters such as disease site, disease stage and LDH cutoff, and all data were limited to multivariate analyses. Disease site subgroups were generated if at least three studies on that site were available; the remaining studies were pooled in a subgroup termed “other.” LDH cutoff subgroups were < 250 U/L, 250–300 U/L, 301–400 U/L, and >400 U/L. In three studies, the effect of LDH was reported as a continuous variable; we pooled those studies separately. Univariate meta-regression model analysis was performed to evaluate the relationship between covariates (LDH cutoff) and the HR for OS.

### Statistical analyses

The meta-analysis was performed with RevMan 5.2 analysis software (Cochrane Collaboration, Copenhagen, Denmark). Estimates of HRs were weighted and pooled using the generic inverse-variance and random-effect model^16^. Analyses were conducted for all studies, and differences between the subgroups were assessed using methods described by Deeks et al.^17^. Publication bias was assessed by visual inspection of the funnel plot. Heterogeneity was assessed using Cochran Q and I^2^ statistics. Meta-regression analysis was conducted using Stata12.0 software. All statistical tests were two-sided, and statistical significance was defined as P less than 0.05. No correction was made for multiple testing.

## Results

### Description of studies

Sixty-eight studies were included in the meta-analysis. The selection process for the systematic review is shown in Figure S1 and the characteristics of the included studies are shown in Table 1. A total of 31,857 patients were included and the median trial sample size was 363.

### Overall survival

Sixty-three studies comprising 29,620 patients reported HRs for OS. All studies analyzed LDH as a dichotomous variable. The studies have clearly shown that upper limit of normal (ULN) remains common for high LDH. The median cutoff for high LDH was 250U/L (range = 200–1000).

**Figure 1:**
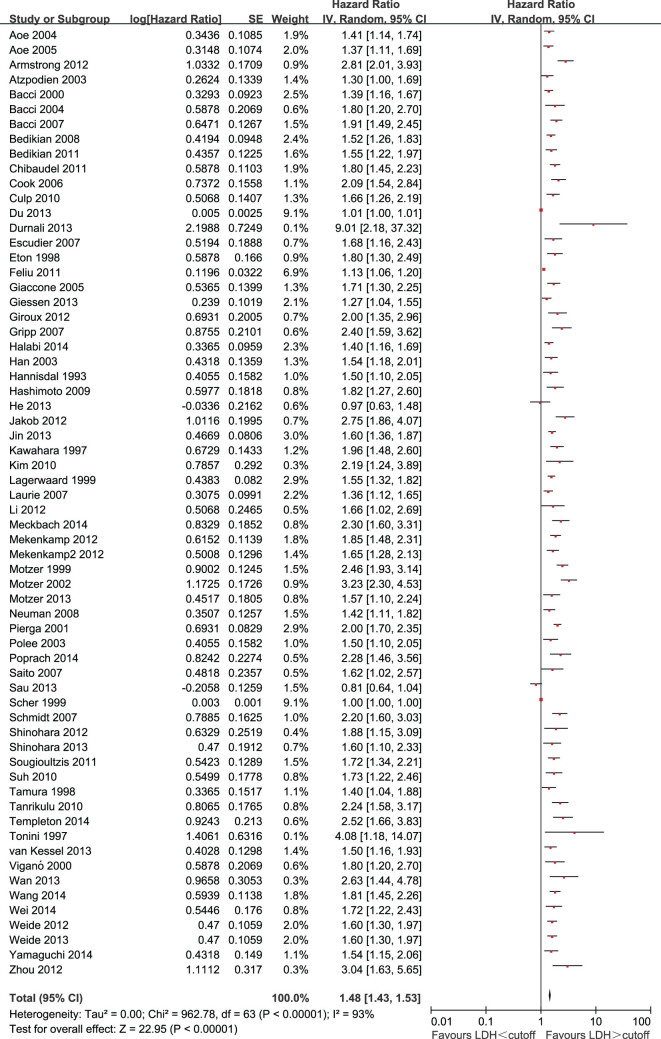
Forest plots showing HR for OS for LDH greater than or less than the cutoff. HRs for each study are represented by the squares, the size of the square represents the weight of the study in the meta-analysis, and the horizontal linecrossing the square represents the 95% confidenceinterval (CI). All statistical tests were two-sided.

Two of the 63 eligible studies (3.2%) reported a non-statistically significant HR. A forest plot of all studies is presented in Figure 1. Overall, LDH greater than the cutoff was associated with a HR for OS of 1.48 (95% CI = 1.43 to 1.53; P < 0.00001). As the heterogeneity among studies was significant (P < 0.00001; I^2^ = 93%), a random-effects model was applied. To explore potential sources of heterogeneity, we performed subgroup analysis in the following subgroups: disease site, tumor stage, and LDH subdivided by predefined cutoffs.

**Figure 2:**
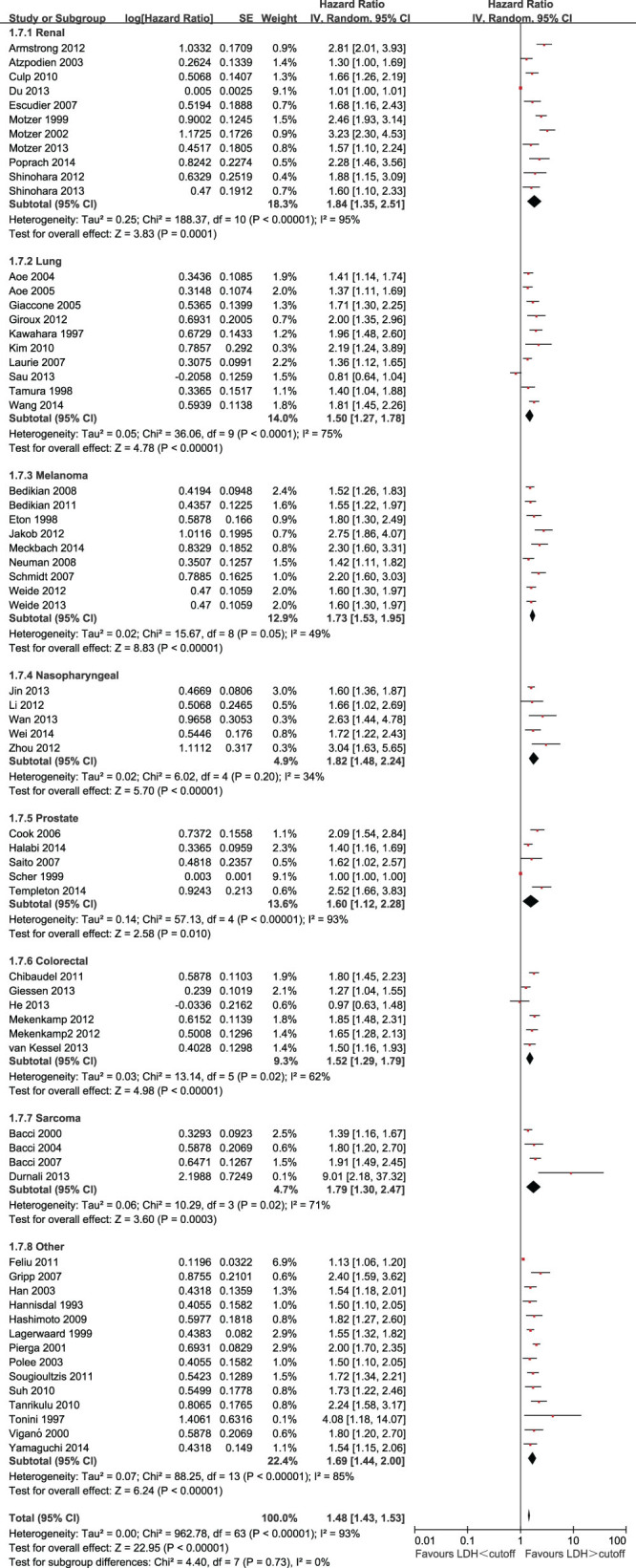
Forest plots showing HRs by disease subgroups.

The effect of LDH on OS among disease subgroups is shown in Figure 2. The prognostic effect of LDH was highest in renal cell carcinoma (HR = 1.84, 95% CI = 1.35 to 2.51), followed by nasopharyngeal carcinoma (HR = 1.82, 95% CI = 1.48 to 2.24), sarcoma (HR = 1.79, 95% CI = 1.30 to 2.47), melanoma (HR = 1.76, 95% CI = 1.56 to 1.98), prostate cancer (HR = 1.55, 95% CI = 1.06 to 2.26), colorectal cancer (HR = 1.52, 95% CI = 1.29 to 1.79), and lung cancer (HR = 1.50, 95% CI = 1.27 to 1.78). The HR for the subgroup of other unselected solid tumors was 1.69 (95% CI = 1.44 to 2.00). For the eight disease-site subgroups analyzed, there was statistically significant heterogeneity between disease sites (P < 0.00001), but no significant differences in the prognostic values of LDH between the subgroups (P for subgroup difference = 0.68).

**Figure 3:**
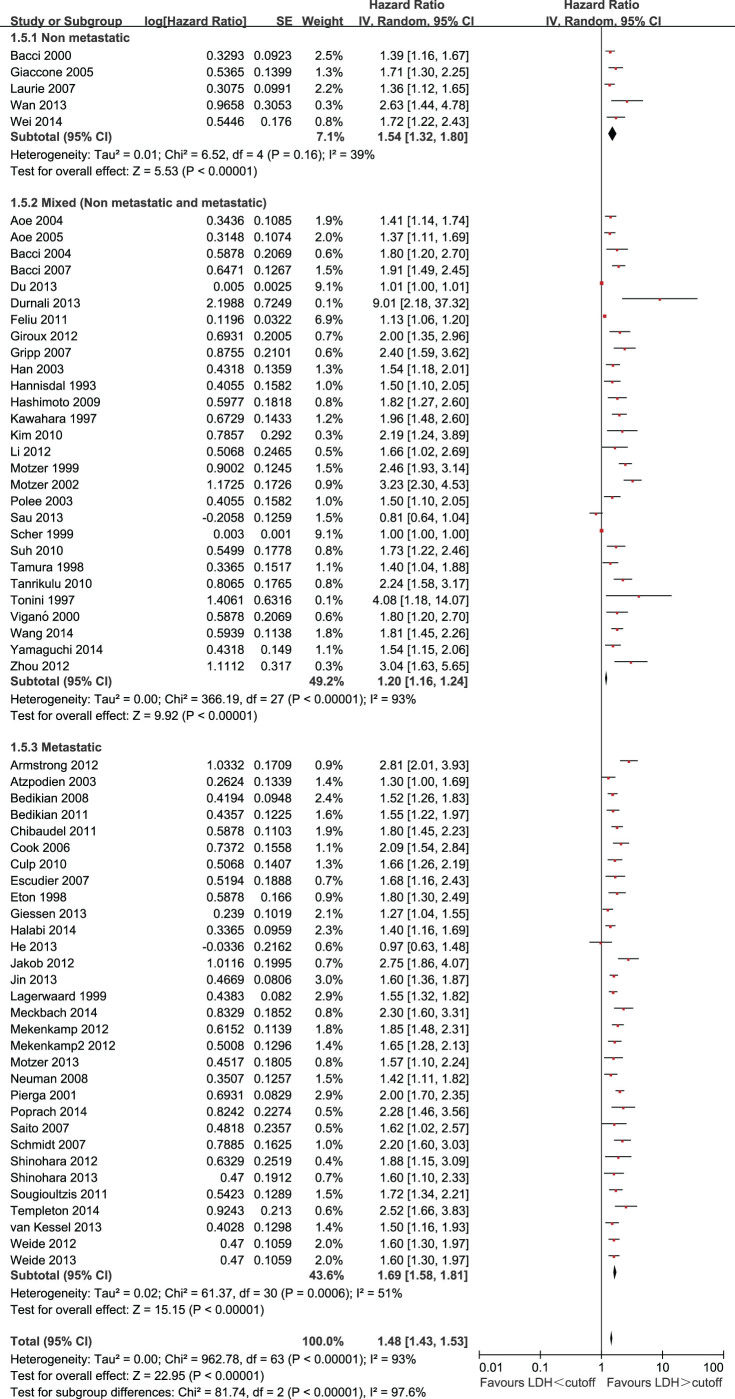
Forest plots showing HRs by stage subgroups.

The effect of LDH on OS among different disease stages is shown in Figure 3. The HRs were 1.54 (95% CI = 1.32 to 1.80) for non-metastatic disease, 1.70 (95% CI = 1.59 to 1.82) for metastatic disease, and 1.20 (95% CI = 1.16 to 1.24) for a mixed group consisting of studies that included both metastatic and non-metastatic patients. There was statistically significant heterogeneity between disease stages (P < 0.00001). The prognostic value of LDH also varied significantly between different disease stages (P for subgroup difference < 0.00001).

**Figure 4:**
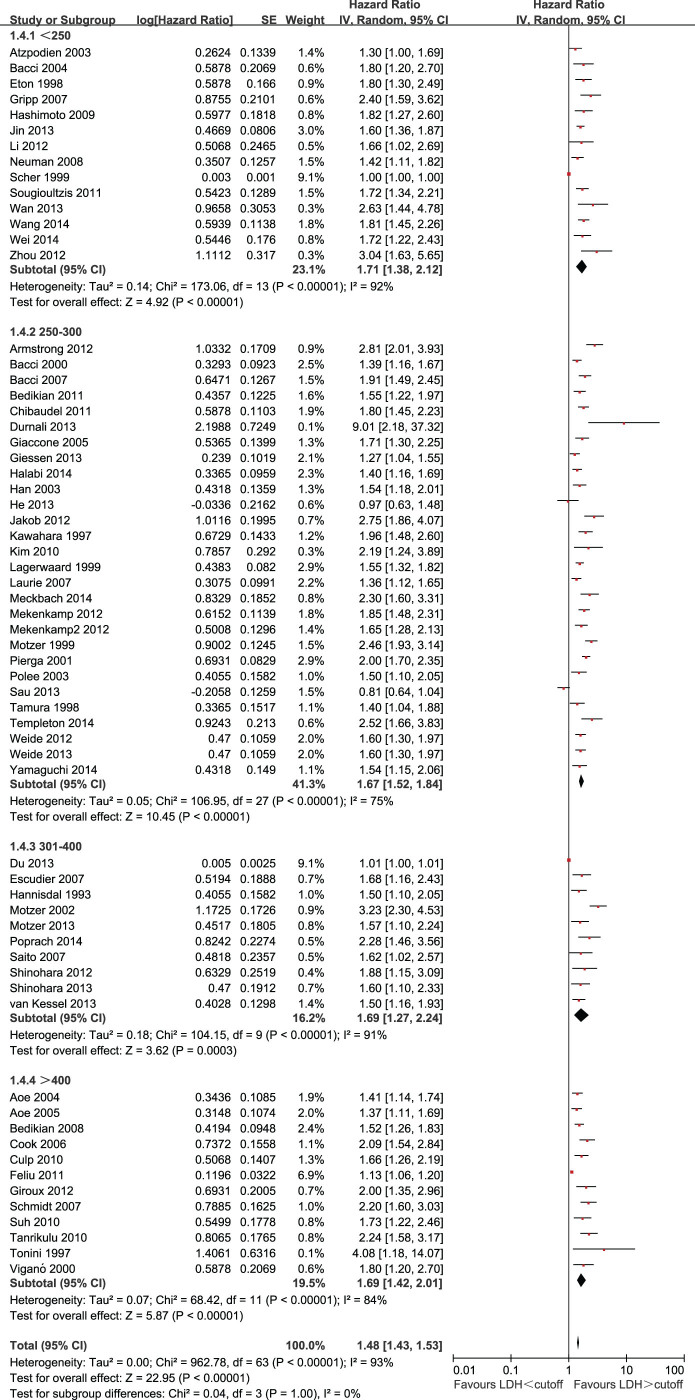
Forest plots showing HRs by LDH cutoffs.

The effect of LDH on OS among different cutoffs for LDH is shown in Figure 4. The HRs were 1.71 (95% CI = 1.38 to 2.12) for LDH cutoff < 250U/L, 1.67(95% CI = 1.52 to 1.84) for LDH cutoff 250 to 300U/L, 1.69 (95% CI = 1.27 to 2.24) for LDH cutoff 301 to 400U/L, and 1.72(95% CI = 1.45 to 2.05)for LDH cutoff > 400 U/L. There was no statistically significant heterogeneity between the different cutoffs for LDH (P for subgroup difference = 0.99).

**Figure 5:**
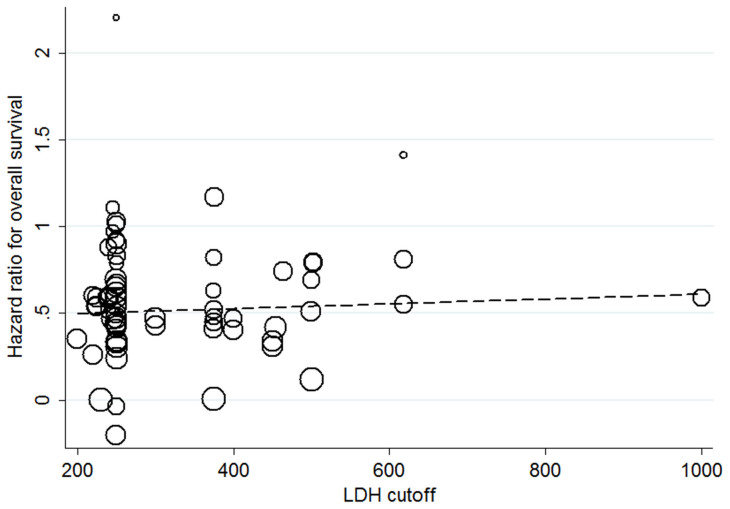
Study-level (i.e., at the individual publication level) association of the cutoff used to define LDH and the HR for overall survival. Each study is represented by a circle, and the area of the circleis proportional to the number of patients enrolled in each study. The gradient of the dashed line represents the results of the meta-regression (β = 1.000138).

The scatter plot for the univariate meta-regression analysis is shown in Figure 5.A total of 63 studies was included in the meta-regression analysis. Overall, there was no statistically significant association between LDH cutoff and the HR for OS (P = 0.614).

There was evidence of publication bias, with fewer small studies reporting negative results than would be expected (Supplementary Figure S2).

Three studies, comprising 1,766 patients, analyzed LDH as a continuous variable and reported HRs for OS. The pooled summary HR of these studies was 2.11 (95% CI, 1.35–3.28; P = 0.0003; I^2^ = 84%) per incremental LDH unit (Supplementary Figure S5).

### Progression-free survival

Six studies, comprising 2,451 patients, reported HRs for PFS. Overall, LDH greater than the cutoff was associated with a HR for PFS of 1.70 (95% CI = 1.44 to 2.01; P < 0.00001; I^2^ = 13%). A forest plot is presented as Figure S3.

### Disease-free (Recurrence-free) survival

A total of five trials, comprising 1,992 patients, reported HRs for DFS. Overall, LDH greater than the cutoff was associated with a HR for the endpoints of 1.86 (95% CI = 1.15 to 3.01; P = 0.01; I^2^ = 88%). A forest plot is presented in Figure S4.

## Discussion

This is the first comprehensive meta-analysis of the prognostic relevance of LDH in solid tumors and it is based on a large pool of clinical studies (31,857 patients). We found a consistent effect of an elevated LDH on OS (HR = 1.48, 95%CI = 1.43 to 1.53) across all disease subgroups and stages. In addition, there is a trend toward a stronger prognostic value of LDH in metastatic disease compared with non-metastatic disease, which may reflect greater tumor burden. The prognostic impact of LDH on PFS and DFS (or RFS) is also robust. Interestingly, different cutoffs of LDH for different disease sites were reported in the included studies. However, the result of subgroups analysis for LDH cutoff showed that there was no association between LDH cutoff and reported HR for OS. This result was confirmed by meta-regression of LDH cutoff and HR for OS. Moreover, LDH was also related to poor prognosis in solid tumors when analyzed as a continuous variable. Our conclusions are supported by the fact that our selected studies were confined to those that used proportional hazards modeling to adjust for clinical prognostic factors and where the sample size was greater than 200.

There is a good biologic rationale for the use of LDH as a prognostic marker for cancer patients; however, the exact mechanism is not understood. One potential mechanism may be an association between LDH and the well-established phenomenon of oncogenicanaerobic glycolysis, or the Warburg effect^5^. This metabolic reprogramming is regulated by HIF-1α, as well as myc, through the transcriptional activation of key genes encoding metabolic enzymes; these include LDH, which converts pyruvate to lactate. This process is closely associated with an increased risk of invasion, metastasis, and patient death^77^.

These analyses have several important implications. First, they show that a high LDH is associated with worse outcome, which suggests that LDH may be a useful biomarker to direct therapeutic selection^78^,^79^.This is because LDH is under the translational control of HIF-1α, as well as myc, and thus is regulated by key oncogenic processes, such as the phosphatidylinositol 3-kinase/Akt/TORC1/hypoxia-inducible factor (PI3K/Akt/TORC1/HIF) pathway^80^,^81^,^82^. A recent study has demonstrated that the TORC1 inhibitor, temsirolimus, could provide therapeutic benefit in patients with RCC and high LDH^79^. Further work to investigate the predictive value of pretreatment LDH in other solid tumors may provide a more general insight into which patients derive benefit from TORC1 inhibition. Second, they show that increased LDH may be interpreted as reflecting high tumor burden or tumor aggressiveness. This suggests that dynamic changes of LDH level may be useful for predicting the prognosis in cancer patients after a primary operation, adjuvant chemotherapy, hormonal therapy, or radiotherapy^65^. Third, LDH allows the identification of a subgroup of tumors with a worse outcome. It is essential in the treatment of cancer to distinguish between low- and high-risk patients, thereby allowing stratification for standard or intensified treatment protocols. It has been shown that LDH can be used as an effective biomarker to guide the selection of regorafenib in patients with colorectal cancer; patients with high LDH may not be optimal candidates for regorafenib^83^.To adequately address these issues and dissect the complex relationship between LDH and cancer, future studies should be conducted within tumor- and stage-specific cohorts.

The strengths of this meta-analysis include the large sample size, estimation of HR using multivariate proportional hazards modeling that adjusted for clinical prognostic factors, and analysis of a massive dataset comprising a large pool of clinical studies. LDH is also likely to be a cancer-specific biomarker, given that it is rarely increased in patients without cancer^84^. Thus, LDH may be a universal prognostic marker in cancer. To improve research in this area, studies with a more specific focus, such as those that address the impact of an individual LDH level on the prognosis of a homogeneous population of cancer patients (i.e., patients with the same cancer stage and subtype), would likely be more informative.

These analyses have limitations. One of the main limitations is the significant heterogeneity between studies, although we used random-effects models when pooling subgroup data. The heterogeneity in these studies could be explained by different patient characteristics or study designs. To facilitate interpretation, we grouped the patients by tumor type and tumor stage. Another limitation is that this is a literature-based analysis. It is compromised by the potential for publication bias, in which there is a tendency for predominantly positive results to have been published, thus inflating our estimate for the association between LDH and outcome. Our strict inclusion criteria (study size greater than 200, the requirement for HRs, and a requirement for a 95% CI or P value) may have introduced selection bias. Most of the included studies were retrospective, which may have introduced reporting bias. Finally, different cutoffs used to assess high LDH level in these studies might also have contributed to the heterogeneity because it is possible that more false-positive cases were obtained with a cutoff of < 300 U/L than with a cutoff of >300 U/L. However, there is no accepted and validated absolute LDH level above which high LDH can be assigned. Instead, we used a cutoff of ULN. This may have introduced substantial heterogeneity, which may not have been fully accounted for by our use of sensitive analyses. The use of ULN is less robust; however, this was the only feasible method with the data available. An internationally accepted and validated LDH cutoff is warranted.

In summary, our data suggest that pretreatment LDH is a simple, cost-effective prognostic factor that can be considered as a criterion to consider patients in different prognostic groups. LDH is also a potential predictive marker to guide individual therapy decisions in solid tumors. Further, adequate, multi-center prospective studies are required to explore the clinical utility of LDH in solid tumors.

## Supplementary Material

Supplementary Information Supplementary Information

## Figures and Tables

**Table 1:** Baseline Characteristics of Included Studies

No	Fist Author	Year	Sample Size	LDH (High/Low)	Site	Stage	Cutoff (UI/L)	Outcome	Study type	Follow-up Time(mo)	Risk of Bias	Adjusted Variable
1	Laurie^41^	2007	210	109/47	SCLC	N	ULN	OS	P	NA	L	Gender, ECOG PS, Anemia grade
2	Motzer^7^	2013	1059	NA	RCC	M	1.5ULN	PFS/OS	R	NA	L	Ethnic origin, ECOG PS, Time from diagnosis to treatment, Bone metastases, Hb, Ca, Neutrophils, Cytokine
3	Polee^32^	2003	350	296/54	Esophageal cancer	M + N	ULN	OS	R	NA	L	WHO Performance, Extent of disease, Paclitaxel
4	Han^31^	2003	383	232/151	Many kinds of cancer	M + N	ULN	OS	R	NA	H	PS(WHO), White blood count, Hb, Number of sites of metastases
5	Atzpodien^30^	2003	425	330/95	RCC	M	220	OS	R	20 +	L	Neutrophil counts, CRP, Time from diagnosis of tumour to metastatic disease, Number of metastatic sites, Bone metastases
6	Bidard^56^	2012	267	121/99	Breast cancer	M	ULN	PFS	P	14.9	L	Triple negative, PS, Number of metastatic sites, CTC, CA15-3, CYFRA 21-1, CEA,ALP, C-INDEX
7	Culp^47^	2010	566	107/366	RCC	M	618	OS	P	20	L	Albumin, ALP, Hb, Metastasectomy at any time, Liver metastasis, Clinical tumor classification, Fuhrman nuclear grade, No. of metastatic sites at CN, Sarcomatoid dedifferentiation, Clear cell histology, treatment
8	Pierga^28^	2001	1336	1039/297	Breast cance	M	ULN	OS	P	NA	L	Karnofsky index, Disease free interval, No. of metastatic sites, Liver involvement, Adjuvant chemotherapy
9	Cook^37^	2006	635	566/69	HRPC	M	454	OS	R	NA	L	Age, PSA, Hb, Albumin, Analgesics, ECOG, NTx, BAP
10	Wan^8^	2013	400	367/33	Nasopharyngeal carcinoma	N	245	DFS/OS	R	NA	L	Age, Tumor stage, Node stage
11	Mekenkamp^9^	2012	1010	637/365	Colorectal cancer	M	ULN	OS	R	NA	L	Diameter, Invasion depth, Lymph node status, Number lymph nodes, Number positive lymph nodes, MMR status, KRAS mutation status,BRAF mutation status
12	Sougioultzis^54^	2011	311	137/173	Gastric carcinoma	M	225	OS	R	NA	L	Palliative gastrectomy, Chemotherapy, Liver metastasis, Abdominal/Peritoneal metastasis, Histological grade, CA72−4, Weight loss, Blood transfusions
13	Zhou^61^	2012	465	424/31	Nasopharyngeal carcinoma	M + N	245	DFS/OS	R	44.7	L	N category, T category, Age
14	Lagerwaard^23^	1999	1292	1081/211	Many kinds of cancer	M	ULN	OS	R	NA	L	PS, Number and distribution of brain metastases, Site of primary tumor, Histology, Interval between primary tumor and brain metastases, Systemic tumor activity, Response to steroid treatment, Treatment modality
15	Aoe^35^	2005	309	448/157	Lung cancer	M + N	450	OS	R	NA	H	Anemia, TNM stage ECOG PS, Sex, Histologic type, Age
16	Bacci^38^	2007	742	464/278	Ewing’s sarcoma	M + N	ULN	OS	R	NA	L	Pelvis, Other sites, Interval symptoms to diagnosis, Fever
17	Armstrong^55^	2012	404	264/140	RCC	M	ULN	OS	R	NA	H	Treatment, Interaction term, KPS, Prior nephrectomy, No. of metastatic sites, Corrected calcium, Hb
18	Gripp^40^	2007	205	130/75	Many kinds of cancer	M + N	240	OS	P	NA	L	WBC, Dyspnea,Morphine, KPS, Brain metastasis, Colorectal, Breast
19	Giaccone^36^	2005	216	NA	SCLC	N	ULN	OS	P	NA	H	Sex, Chest radiotherapy, PCI, Platelets
20	Motzer^29^	2002	463	NA	RCC	M + N	1.5 ULN	OS	R	46	L	Karnofsky PS, Hb, Calcium, Time from initial RCC diagnosis to start of interferon-alpha therapy
21	Bacci^26^	2000	357	238/121	Ewing’s sarcoma	N	ULN	OS	R	126	L	Sex, Age, Fever, Anemia, Axial location, Radiation therapy only for local control, Type of chemotherapy regimen, Chemotherapy-induced necrosis
22	Motzer^24^	1999	670	NA	RCC	M + N	1.5ULN	OS	R	33	L	KPS, Hb, Ca, Prior nephrectomy.
23	Feliu	2011	406	NA	Many kinds of cancer	M + N	NA	OS	P	NA	H	ECOG PS, TTD, Albumin, Lymphocytes
24	Scher^25^	1999	254	164/90	CRPC	M + N	230	OS	R	NA	H	No 50% decline within 12 wk, Hb, Age
25	Escudier^39^	2007	300	222/52	RCC	M	1.5ULN	OS	P	NA	L	ECOG PS, Number of metastatic sites, Time from nephrectomy to metastatic disease, ALP, Ca
26	Kawahara^19^	1997	284	147/137	SCLC	M + N	ULN	OS	R	NA	H	PS, Stage, ALP, CEA, Sex
27	Chibaudel^52^	2011	535	283/252	Colorectal Cancer	M	ULN	OS	R	NA	L	Age, Sex, PS, No.sites, Liver involvement, Primitive tumor, Time to metastasis, Adjuvant CT, ALP, CEA
28	Kim^48^	2010	257	NA	NSCLC	M + N	ULN	OS	R	NA	H	ECOG PS, Skin rash
29	Hashimoto^46^	2009	326	NA	Pancreatic cancer	M + N	220	OS	R	NA	H	Recurrence vs. metastasis, KPS, Liver metastasis, Peritoneal metastasis, ALP, CRP
30	Tanrikulu^50^	2010	363	NA	Pleural mesothelioma	M + N	500	OS	R	NA	H	KPS, Pleural fluid glucose level, CRP, Pleural effusion, Pleural thickening on chest CT, Platelet count
31	Aoe^33^	2004	611	NA	Lung Cancer	M + N	450	OS	R	NA	H	Platelet count, TNM stage, ECOG PS, Sex, Histologic type, Age
32	Giroux^10^	2012	245	177/45	NSCLC	M + N	500	OS	R	NA	H	Number of treatment lines, PS, Surgery, Maintenance therapy, Time to first progression of tumour
33	Suh^49^	2010	209	94/115	Many kinds of cancer	M + N	502	OS	R	NA	H	Anorexia, Resting dyspnea, ECOG, Leukocytosis, Bilirubin, Creatinine
34	Bacci^34^	2004	1421	1116/305	Osteosarcoma	M + N	240	OS	R	NA	L	Other sites, Interval symptoms to diagnosis, Treatment
35	Saito^42^	2007	241	NA	Prostate Cancer	M	400	OS	R	31	L	Age, performance status, clinical presentation, disease localization, pathologic findings, PSA, PSA/PAP ratio, CEA, ALP,CRP
36	Hannisdal^18^	1993	202	NA	Bladder Cancer	M + N	400	OS	R	NA	H	Erythrocyte sedimentation rate, Hb, ALP, GGT, Creatinine, Albumin
37	Tonini^20^	1997	246	162/106	Neuroblastoma	M + N	1000	OS	R	NA	L	MYCN oncogene amplification, Abdominal tumor, Stage, Vanillylmandelic (VMA) urinary excretion, Ferritin, Neuron-specific enolase (NSE)
38	Li^58^	2012	533	NA	Nasopharyngeal carcinoma	M + N	240	OS	R	NA	H	AJCC T category, AJCC N category, Age
39	Jin^65^	2013	689	379/310	Nasopharyngeal carcinoma	M	245	OS	R	NA	L	Sex, Age, Metastasis at presentation, Lung metastasis, Post-treatment S-LDH level, Drug number of chemotherapy, Number of involved sites, Liver metastasis, Bone metastasis
40	Wei^75^	2014	601	NA	Nasopharyngeal carcinoma	N	225	DFS/OS	R	51.5	L	Age, T classification, N classification
41	Sau^14^	2013	329	154/175	NSCLC	M + N	ULN	OS	R	NA	L	Age, Sex, PS, Histopathology, smoking status, Response after 1-line CT, First-line CT, PFS after 1-line CT, Second-line CT
42	Wang^74^	2014	499	75/39	SCLC	M + N	240	OS	R	NA	L	ECOG-PS, Extensive disease, NLR
43	Yamaguchi^76^	2014	206	NA	Neuroendocrine carcinoma of the digestive system	M + N	ULN	OS	R	NA	H	Age, Sex, PS, Primary site, Liver metastasis, First-line chemotherapy, Prior surgery
44	Halabi^70^	2014	1050	565/482	CRPC	M	ULN	OS	R	NA	L	ECOG PS, Disease site, Opioid analgesic use, Albumin, Hb, PSA,ALP
45	Templeton^73^	2014	357	NA	CRPC	M	1.2 ULN	OS	R	NA	H	Age, ECOG PS, Number of comorbidities, Gleason sum score, Lymph node metastatic only, Bone metastasis, Visceral metastasis, Liver metastasis, Hb, Albumin, ALP, PSA, PSA-doubling time, NLR
46	Du^62^	2013	286	197/89	RCC	M + N	1.5 ULN	DFS/OS	R	NA	L	Fibrinogen, Hb, Ca, T stage, Fuhrman grade, Tumor size
47	Shinohara^67^	2013	473	388/34	RCC	M	1.5 ULN	OS	R	NA	L	Time from initial diagnosis to metastasis, Hb, Ca, CRP, Liver metastasis, Bone metastasis, Lymph node metastasis
48	Poprach^72^	2014	319	285/34	RCC	M	1.5 ULN	PFS/OS	R	15	L	Time from diagnosis to TKI, Neutrophils, ECOG PS
49	Powles^66^	2013	204	52/55	Seminoma	M + N	1.5 ULN	PFS	R	NA	H	Age, IPFSG score
50	van Kessel^68^	2013	290	152/138	Colorectal Cancer	M	ULN	OS	R	NA	L	Gender, Age, Number of first line cycles, Metastases, Resection prim. Tumour, Study-arm, Response category
51	Giessen^64^	2013	215	270/201	Colorectal Cancer	M	250	OS	R	55.4	L	Liver-limited disease, N-stage of primary, KPS, ALP
52	Weide^69^	2013	372	263/175	Melanoma	M	ULN	OS	R	27	L	S100B, Cerebral metastases, First systemic therapy
53	Meckbach^71^	2014	215	131/63	Melanoma	M	ULN	OS	R	46	L	Brain metastasis
54	Durnali^63^	2013	240	101/81	Osteosarcoma	M + N	ULN	RFS/OS	R	51	L	Gender, ALP, Histological subtype, Metastasis at diagnosis, Surgical margins, Tumor necrosis rate, Postoperative chemotherapy, Surgery after recurrence, Chemotherapy after recurrence,,
55	He^13^	2013	239	154/82	Colorectal Cancer	M	ULN	PFS/OS	R	NA	H	Age, Gender, Lines of chemotherapy,CEA,CA19-9, GGT,ALP
56	Weide^60^	2012	855	502/228	Melanoma	M	ULN	OS	R	NA	L	S100B, Time interval between initial diagnosis and stage IV diagnosis, Site of distant metastasis, Number of involved distant sites
57	Shinohara^59^	2012	361	299/23	RCC	M	1.5ULN	OS	R	21.5	L	Time from initial diagnosis to treatment, Hb, Prognostic metastatic group
58	Jakob^57^	2012	677	263/97	Melanoma	M	ULN	OS	R	12	L	Age, Gender, M1 Category, Mutation
59	Bedikian^51^	2011	740	430/275	Melanoma	M	ULN	OS	R	NA	L	Age, Chemoresponse, Albumin, M-stage, Location of primary melanoma
60	Neuman^45^	2008	589	246/125	Melanoma	M	200	OS	P	NA	L	Sex, Age at diagnosis of stage IV disease, Antecedent stage, DFI, Site of disease, No. of organs involved, No. of metastases
61	Schmidt^43^	2007	363	317/46	Melanoma	M	2ULN	PFS/OS	R	50.4	L	Sex, Site, ECOG PS, Leukocytes, Neutrophils
62	Bedikian^44^	2008	616	358/258	Melanoma	M	618	OS	R	NA	L	ECOG PS, Disease stage, Metastatic sites, Visceral metastasis, Albumin, Response to treatment
63	Viganó^27^	2000	227	142/85	Many kinds of cancer	M + N	618	OS	R	NA	L	Primary tumor, Liver metastasis, Comorbidity, Weight loss, ECOG PS, Nausea, Clinical estimation of survival, Albumin, Lymphocyte count
64	Tamura^22^	1998	253	NA	SCLC	M + N	ULN	OS	R	NA	H	Extent of disease, Number of metastatic sites, Albumin, Weight loss
65	Eton O^21^	1998	318	NA	Melanoma	M	225	OS	R	NA	H	Albumin, Soft tissue and/or single visceral organ metastases (especially lung), Sex, Enrollment late in the decade
66	D’AMICO^77^	2005	494	NA	HRPC	M	74-2077	OS	R	15.6-16.8	L	Hb, Age, ECOG PS, ALP, Treatment, PSA response duration, PSA
67	Halabi^78^	2003	760	NA	HRPC	M	173-437	OS	R	NA	H	PS, Gleason, ALP, PSA, Visceral disease, Hb
68	Schellhammer^79^	2013	512	NA	CRPC	M	84-1662	OS	P	NA	L	PSA, Hb, ECOG, ALP, Gleason score

Abbreviations: SCLC: small-cell lung cancer; NSCLC: non-small-cell lung cancer; RCC: renal cell carcinoma; HRPC: hormone-refractory prostate cancer; CRPC: castration refractory prostate cancer; ULN: upper limit of normal; OS: overall survival; PFS: progression-free survival; DFS: disease-free survival; RFS: recurrence-free survival; M: metastatic; N: non-metastatic; M + N: mixed (non-metastatic and metastatic); R: retrospective; P: prospective; L : low risk; High: high risk; NA: not available; PS: performance score; KPS: Karnofsky performance score ; LDH : Lactic dehydrogenas; ALP: alkaline phosphatase; PSA: prostate specific antigen; Hb: hemoglobin; Ca: calcium; PS: Performance Status; ECOG PS: Eastern Cooperative Oncology Group Performance Status ; ALP: alkaline phosphatase; CTC: circulating tumor; NLR: neutrophils / lymphocytes; CRP: C-reaction protein; IPFSG: International Prognostic Factors Study Group; CA19-9: carbohydrate antigen 19-9; CEA: carcinoembryonic antigen; GGT: gamma-glutamyl transpeptidase; DFI: DFI: disease-free interval
