# Supplementary material for: Prognostic value of pretreatment serum lactate dehydrogenase level in patients with solid tumors: a systematic review and meta-analysis
Source: Sci Rep. 2015 Apr 22;5:9800. doi: 10.1038/srep09800 (PMC5386114; doi:10.1038/srep09800)
Supplement: Supplementary Information [file srep09800-s1.pdf]

Prognostic value of pretreatment serum lactate dehydrogenase level in patients with solid tumors: a systematic review and meta-analysis

Jiao Zhang, Yan-Hong Yao, Bao-Guo Li, Qing Yang, Peng-Yu Zhang, Hai-Tao Wang*

Supplements:

Figure:

S1. Flow diagram showing the selection process for the systematic review.

S2. Publication Bias (Funnel plot of hazard ratio for overall survival for Lactate dehydrogenase (LDH) (horizontal axis) and the standard error (SE) for the hazard ratio (vertical axis). Each study is represented by one circle. The vertical line represents the pooled effect estimate.)

S3. Forest plots showing hazard ratio for progression-free survival for Lactate dehydrogenase (LDH) greater than or less than the cutoff.

S4. Forest plots showing hazard ratio for disease-free survival/recurrence-free survival for Lactate dehydrogenase (LDH) greater than or less than the cutoff.

S5. Forest plots showing hazard ratio for overall survival for LDH as a continuous variable.

Figure: Risk of bias summary

Other supplements material:

S1-Documentation of the Searches

S2-Excluded References

Results of Meta-regression

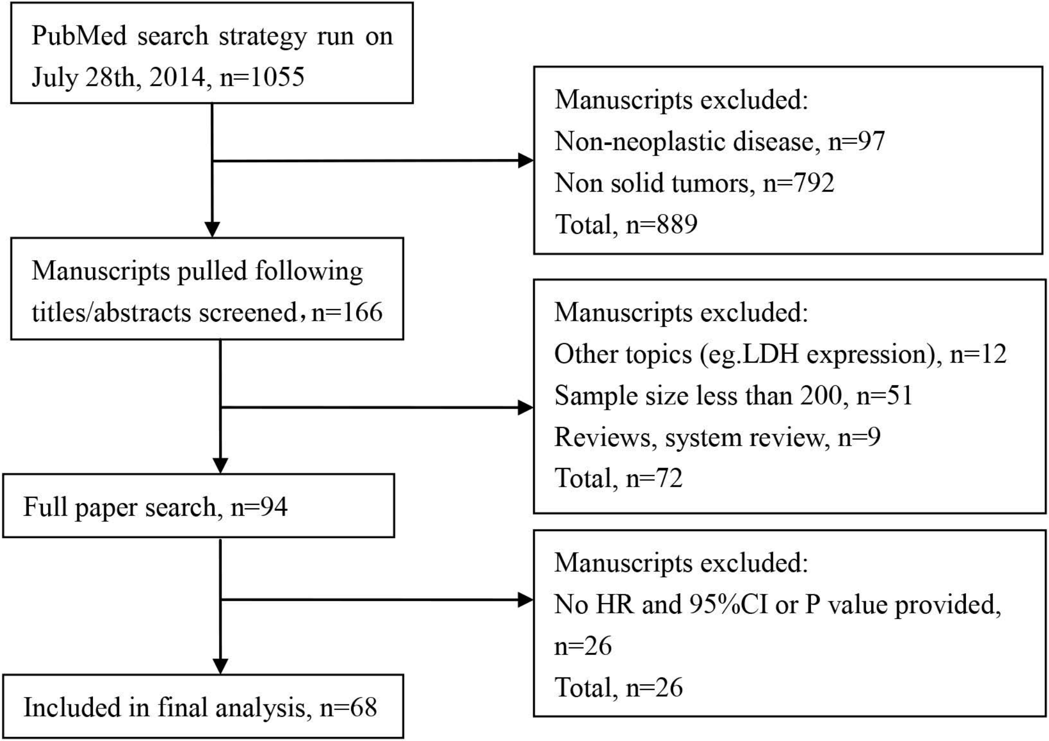
PubMed search strategy run on July 28th, 2014, n=1055
Manuscripts excluded:
Non-neoplastic disease, n=97
Non solid tumors, n=792
Total, n=889
Manuscripts pulled following titles/abstracts screened，n=166
Manuscripts excluded:
Other topics (eg.LDH expression), n=12
Sample size less than 200, n=51
Reviews, system review, n=9
Total, n=72
Full paper search, n=94
Manuscripts excluded:
No HR and 95%CI or P value provided, n=26
Total, n=26
Included in final analysis, n=68

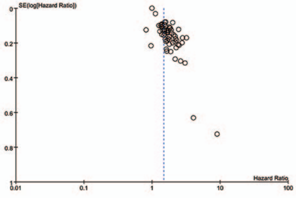
SE(log[Hazard Ratio])
0
0.2
0.4
0.6
0.8
1
Hazard Ratio
0.01
0.1
1
10
100

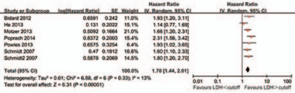
Hazard Ratio
Hazard Ratio
Study or Subgroup log[Hazard Ratio] SE Weight IV, Random, 95% CI
IV, Random, 95% CI
Bidard 2012 0.6591 0.242 11.0% 1.93 [1.20, 3.11]
He 2013 0.131 0.2022 15.1% 1.14 [0.77, 1.69]
Motzer 2013 0.5092 0.1664 21.0% 1.66 [1.20, 2.31]
Poprach 2014 0.8372 0.2003 15.4% 2.31 [1.56, 3.42]
Powles 2013 0.6575 0.3254 6.4% 1.93 [1.02, 3.65]
Schmidt 2007 0.47 0.1912 16.6% 1.60 [1.10, 2.33]
Schmidt2 2007 0.5878 0.2069 14.5% 1.80 [1.20, 2.70]
Total (95% CI) 100.0% 1.70 [1.44, 2.01]
Heterogeneity: Tau² = 0.01; Chi² = 6.88, df = 6 (P = 0.33); I² = 13%
Test for overall effect: Z = 6.31 (P < 0.00001)
0.01
0.1
1
10
100
Favours LDH<cutoff
Favours LDH>cutoff

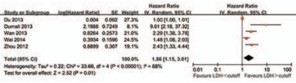

Hazard Ratio
Study or Subgroup
log[Hazard Ratio]
SE
Weight
IV, Random, 95% CI
Du 2013 0.004 0.002 27.3% 1.00 [1.00, 1.01]
Durnali 2013 2.1988 0.7249 8.1% 9.01 [2.18, 37.32]
Wan 2013 0.8264 0.2573 21.0% 2.29 [1.38, 3.78]
Wei 2014 0.3934 0.1596 24.5% 1.48 [1.08, 2.03]
Zhou 2012 0.8899 0.307 19.1% 2.43 [1.33, 4.44]
Total (95% CI) 100.0% 1.86 [1.15, 3.01]
Heterogeneity: Tau² = 0.22; Chi² = 33.66, df = 4 (P < 0.00001); I² = 88%
Test for overall effect: Z = 2.52 (P = 0.01)
Hazard Ratio
IV, Random, 95% CI
0.01
0.1
1
10
100
Favours LDH<cutoff
Favours LDH>cutoff

Study or Subgroup	log[Hazard Ratio]	SE	Weight	Hazard Ratio IV, Random, 95% CI	Hazard Ratio IV, Random, 95% CI
D'Amico 2005	0.8755	0.275	21.4%	2.40 [1.40, 4.11]	
D'Amico2 2005	1.1314	0.2236	23.9%	3.10 [2.00, 4.80]	
Halabi 2003	0.3148	0.0634	30.5%	1.37 [1.21, 1.55]	
Schellhammer 2013	0.7921	0.2188	24.2%	2.21 [1.44, 3.39]	
**Total (95% CI)**			**100.0%**	**2.11 [1.35, 3.28]**	

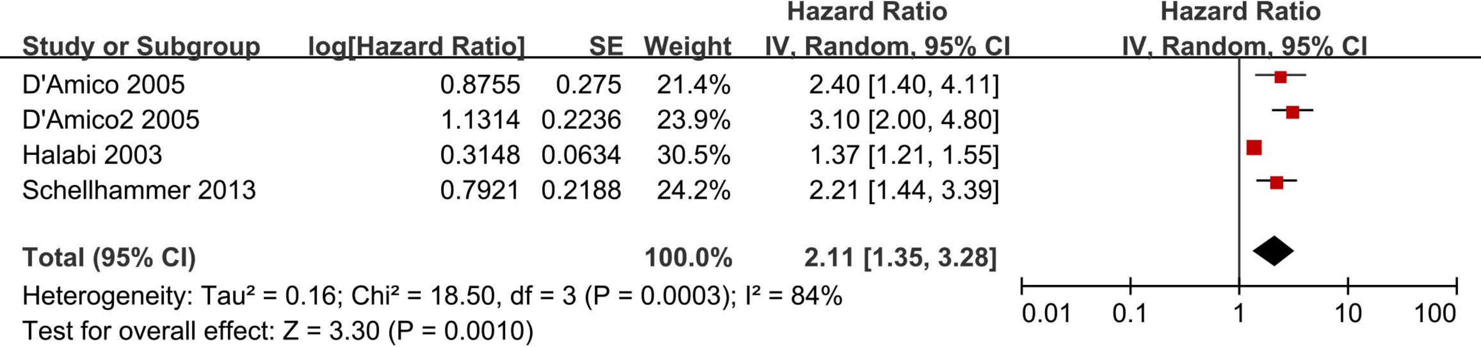

Heterogeneity: $Tau^2 = 0.16$; $Chi^2 = 18.50$, df = 3 (P = 0.0003); $I^2 = 84\%$

Test for overall effect: Z = 3.30 (P = 0.0010)

Study	Were adequate eligibility criteria developed and applied?	Was the measurement of both exposure and outcome adequate?	Was confounding adequately controlled for?	Was the follow-up complete and adequate in duration?	Are study reports free of suggestion of selective outcome reporting?	Was the study free of other problems that put it at a high risk of bias?	Risk of bias
Aoe 2004	+	+	+	+	+	−	−
Aoe 2005	+	+	+	+	+	−	−
Armstrong 2012	+	+	+	+	+	−	−
Atzpodien 2003	+	+	+	+	+	+	+
Bacci 2000	+	+	+	+	+	+	+
Bacci 2004	+	+	+	+	+	+	+
Bacci 2007	+	+	+	+	+	+	+
Bedikian 2008	+	+	+	+	+	+	+
Bedikian 2011	+	+	+	+	+	+	+
Bidard 2012	+	+	+	+	+	+	+
Chibaudel 2011	+	+	+	+	+	+	+
Cook 2006	+	+	+	+	+	+	+
Culp 2010	+	+	+	+	+	+	+
D'Amico 2005	+	+	+	+	+	+	+
Du 2013	+	+	+	+	+	+	+
Durnali 2013	+	+	+	+	+	+	+
Escudier 2007	+	+	+	+	+	+	+
Eton 1998	+	+	+	+	+	−	−
Feliu 2011	+	+	+	+	+	−	−
Giaccone 2005	+	+	+	+	+	−	−
Giessen 2013	+	+	+	+	+	+	+
Giroux 2012	+	+	+	+	+	−	−
Gripp 2007	+	+	+	+	+	+	+
Halabi 2003	+	+	+	+	+	−	−
Halabi 2014	+	+	+	+	+	+	+
Han 2003	+	+	+	+	+	−	−
Hannisdal 1993	+	+	+	+	+	−	−
Hashimoto 2009	+	+	+	+	+	−	−
He 2013	+	+	+	+	+	−	−
Jakob 2012	+	+	+	+	+	+	+
Jin 2013	+	+	+	+	+	+	+
Kawahara 1997	+	+	+	+	+	−	−
Kim 2010	+	+	+	+	+	−	−
Lagerwaard 1999	+	+	+	+	+	+	+
Laurie 2007	+	+	+	+	+	+	+
Li 2012	+	+	+	+	+	+	+
Meckbach 2014	+	+	+	+	+	+	+
Mekenkamp 2012	+	+	+	+	+	+	+
Motzer 1999	+	+	+	+	+	+	+
Motzer 2002	+	+	+	+	+	+	+
Motzer 2013	+	+	+	+	+	+	+
Neuman 2008	+	+	+	+	+	+	+
Pierga 2001	+	+	+	+	+	+	+
Polee 2003	+	+	+	+	+	+	+
Poprach 2014	+	+	+	+	+	+	+
Powles 2013	+	+	+	+	+	−	−
Saito 2007	+	+	+	+	+	+	+
Sau 2013	+	+	+	+	+	+	+
Schellhammer 2013	+	+	+	+	+	+	+
Scher 1999	+	+	+	+	+	−	−
Schmidt 2007	+	+	+	+	+	+	+
Shinohara 2012	+	+	+	+	+	+	+
Shinohara 2013	+	+	+	+	+	+	+
Sougioultzis 2011	+	+	+	+	+	+	+
Suh 2010	+	+	+	+	+	−	−
Tamura 1998	+	+	+	+	+	−	−
Tanrikulu 2010	+	+	+	+	+	−	−
Templeton 2014	+	+	+	+	+	−	−
Tonini 1997	+	+	+	+	+	+	+
van Kessel 2013	+	+	+	+	+	+	+
Viganó 2000	+	+	+	+	+	+	+
Wan 2013	+	+	+	+	+	+	+
Wang 2014	+	+	+	+	+	+	+
Wei 2014	+	+	+	+	+	+	+
Weide 2012	+	+	+	+	+	+	+
Weide 2013	+	+	+	+	+	+	+
Yamaguchi 2014	+	+	+	+	+	−	−
Zhou 2012	+	+	+	+	+	+	+

Pubmed 检索式：

((((((l-lactate dehydrogenase[MeSH Terms]) OR lactate dehydrogenase[Title/Abstract]) OR LDH[Title/Abstract])) AND ((((prognosis[MeSH Terms]) OR prognosis[Title/Abstract]) OR prognoses[Title/Abstract]) OR prognostic[Title/Abstract])) AND ((((((multivariate analysis[MeSH Terms]) OR multivariate analysis[Title/Abstract]) OR proportional hazard model[MeSH Terms]) OR proportional hazard model[Title/Abstract]) OR COX proportional hazard model[Title/Abstract]) OR COX Models[Title/Abstract]))

Run on：2014-7-28

Part 1：

[MeSH Terms]: "l-lactate dehydrogenase"

Title/Abstract："lactate dehydrogenase"

Title/Abstract：“LDH”

Part 2：

[MeSH Terms]: “prognosis”

Title/Abstract：“prognosis”

Title/Abstract：“prognoses”

Title/Abstract：“prognostic”

Part 3：

[MeSH Terms]: “multivariate analysis”

Title/Abstract：“multivariate analysis”

[MeSH Terms]: “proportional hazard model”

Title/Abstract：“proportional hazard model”

Title/Abstract：“COX proportional hazard model”

Title/Abstract：“Cox Models”

Title/abstracts:

Other topics: n=889

Other topics: n=12

[1] Sun X, Sun Z, Zhu Z, et al. Clinicopathological significance and prognostic value of lactate dehydrogenase A expression in gastric cancer patients[J]. PLoS One,2014,9(3):e91068.
[2] Grimm M, Alexander D, Munz A, et al. Increased LDH5 expression is associated with lymph node metastasis and outcome in oral squamous cell carcinoma[J]. Clin Exp Metastasis,2013,30(4):529-540.
[3] Lu R, Jiang M, Chen Z, et al. Lactate dehydrogenase 5 expression in Non-Hodgkin lymphoma is associated with the induced hypoxia regulated protein and poor prognosis[J]. PLoS One,2013,8(9):e74853.
[4] Koukourakis M I, Giatromanolaki A, Sivridis E, et al. Prognostic and predictive role of lactate dehydrogenase 5 expression in colorectal cancer patients treated with PTK787/ZK 222584 (vatalanib) antiangiogenic therapy[J]. Clin Cancer Res,2011,17(14):4892-4900.
[5] Liao A C, Li C F, Shen K H, et al. Loss of lactate dehydrogenase B subunit expression is correlated with tumour progression and independently predicts inferior disease-specific survival in urinary bladder urothelial carcinoma[J]. Pathology,2011,43(7):707-712.
[6] Koukourakis M I, Giatromanolaki A, Winter S, et al. Lactate dehydrogenase 5 expression in squamous cell head and neck cancer relates to prognosis following radical or postoperative radiotherapy[J]. Oncology,2009,77(5):285-292.
[7] Kolev Y, Uetake H, Takagi Y, et al. Lactate dehydrogenase-5 (LDH-5) expression in human gastric cancer: association with hypoxia-inducible factor (HIF-1alpha) pathway, angiogenic factors production and poor prognosis[J]. Ann Surg Oncol,2008,15(8):2336-2344.
[8] Koukourakis M I, Giatromanolaki A, Sivridis E, et al. Lactate dehydrogenase 5 expression in operable colorectal cancer: strong association with survival and activated vascular endothelial growth factor pathway--a report of the Tumour Angiogenesis Research Group[J]. J Clin Oncol,2006,24(26):4301-4308.
[9] von Eyben F E, Blaabjerg O, Madsen E L, et al. Serum lactate dehydrogenase isoenzyme 1 and tumour volume are indicators of response to treatment and predictors of prognosis in metastatic testicular germ cell tumours[J]. Eur J Cancer,1992,28(2-3):410-415.
[10] Yuan C, Li Z, Wang Y, et al. Overexpression of metabolic markers PKM2 and LDH5 correlates with aggressive clinicopathological features and adverse patients' prognosis in tongue cancer[J]. Histopathology,2014.
[11] Kim H S, Lee H E, Yang H K, et al. High lactate dehydrogenase 5 expression correlates with high tumoral and stromal vascular endothelial

growth factor expression in gastric cancer[J]. Pathobiology,2014,81(2):78-85.
[12] Girgis H, Masui O, White N M, et al. Lactate dehydrogenase A is a potential prognostic marker in clear cell renal cell carcinoma[J]. Mol Cancer,2014,13:101.

Reviews: n=9

[1] Donskov F. Interleukin-2 based immunotherapy in patients with metastatic renal cell carcinoma[J]. Dan Med Bull,2007,54(4):249-265.
[2] Nagura E. [Prognositic factors in multiple myeloma][J]. Nihon Rinsho,2007,65(12):2351-2356.
[3] Balch C M, Soong S J, Atkins M B, et al. An evidence-based staging system for cutaneous melanoma[J]. CA Cancer J Clin,2004,54(3):131-149, 182-184.
[4] Galsky M, Kelly W K. Use of nomograms for predicting survival in patients with castrate prostate cancer[J]. Urology,2003,62 Suppl 1:119-127.
[5] Mora J, Gerald W L, Cheung N K. Evolving significance of prognostic markers associated with new treatment strategies in neuroblastoma[J]. Cancer Lett,2003,197(1-2):119-124.
[6] Watine J. Prognostic evaluation of primary non-small cell lung carcinoma patients using biological fluid variables. A systematic review[J]. Scand J Clin Lab Invest,2000,60(4):259-273.
[7] George D J, Kantoff P W. Prognostic indicators in hormone refractory prostate cancer[J]. Urol Clin North Am,1999,26(2):303-310.
[8] Osterlind K. Factors confounding evaluation of treatment effect in lung cancer[J]. Lung Cancer,1994,10 Suppl 1:S97-S103.
[9] Bajorin D F, Geller N L, Bosl G J. Assessment of risk in metastatic testis carcinoma: impact on treatment[J]. Urol Int,1991,46(3):298-303.

Sample size less than 200：n=51

[1] Atkinson B J, Kalra S, Wang X, et al. Clinical outcomes for patients with metastatic renal cell carcinoma treated with alternative sunitinib schedules[J]. J Urol,2014,191(3):611-618.
[2] Cassier P A, Polivka V, Judson I, et al. Outcome of patients with sarcoma and other mesenchymal tumours participating in phase I trials: a subset analysis of a European Phase I database[J]. Ann Oncol,2014,25(6):1222-1228.
[3] Gaudy-Marqueste C, Archier E, Grob A, et al. Initial metastatic kinetics is the best prognostic indicator in stage IV metastatic melanoma[J]. Eur J Cancer,2014,50(6):1120-1124.
[4] Kamba T, Yamasaki T, Teramukai S, et al. Improvement of prognosis in patients with metastatic renal cell carcinoma and Memorial Sloan-Kettering Cancer Center intermediate risk features by modern strategy including molecular-targeted therapy in clinical practice[J]. Int J Clin Oncol,2014,19(3):505-515.

[5] Kang M H, Go S I, Song H N, et al. The prognostic impact of the neutrophil-to-lymphocyte ratio in patients with small-cell lung cancer[J]. Br J Cancer,2014,111(3):452-460.
[6] Shao L, Hong W, Zheng L, et al. [Joint serum tumor markers serve as survival predictive model of erlotinib in the treatment of recurrent non-small cell lung cancer][J]. Zhongguo Fei Ai Za Zhi,2014,17(5):391-400.
[7] Karachaliou N, Papadaki C, Lagoudaki E, et al. Predictive value of BRCA1, ERCC1, ATP7B, PKM2, TOPOI, TOPOmicron-IIA, TOPOIIB and C-MYC genes in patients with small cell lung cancer (SCLC) who received first line therapy with cisplatin and etoposide[J]. PLoS One,2013,8(9):e74611.
[8] Marcus D M, Lowe M, Khan M K, et al. Prognostic Factors for Overall Survival After Radiosurgery for Brain Metastases From Melanoma[J]. Am J Clin Oncol,2013.
[9] Nakagawa T, Hara T, Kawahara T, et al. Prognostic risk stratification of patients with urothelial carcinoma of the bladder with recurrence after radical cystectomy[J]. J Urol,2013,189(4):1275-1281.
[10] Tamiya M, Kobayashi M, Morimura O, et al. Clinical significance of the serum crosslinked N-telopeptide of type I collagen as a prognostic marker for non-small-cell lung cancer[J]. Clin Lung Cancer,2013,14(1):50-54.
[11] Wevers K P, Kruijff S, Speijers M J, et al. S-100B: a stronger prognostic biomarker than LDH in stage IIIB-C melanoma[J]. Ann Surg Oncol,2013,20(8):2772-2779.
[12] Oh J R, Seo J H, Chong A, et al. Whole-body metabolic tumour volume of 18F-FDG PET/CT improves the prediction of prognosis in small cell lung cancer[J]. Eur J Nucl Med Mol Imaging,2012,39(6):925-935.
[13] Fussenich L M, Desar I M, Peters M E, et al. A new, simple and objective prognostic score for phase I cancer patients[J]. Eur J Cancer,2011,47(8):1152-1160.
[14] Pfeil A F, Leiter U, Buettner P G, et al. Melanoma of unknown primary is correctly classified by the AJCC melanoma classification from 2009[J]. Melanoma Res,2011,21(3):228-234.
[15] Richey S L, Culp S H, Jonasch E, et al. Outcome of patients with metastatic renal cell carcinoma treated with targeted therapy without cytoreductive nephrectomy[J]. Ann Oncol,2011,22(5):1048-1053.
[16] Gupta S, Bedikian A Y, Ahrar J, et al. Hepatic artery chemoembolization in patients with ocular melanoma metastatic to the liver: response, survival, and prognostic factors[J]. Am J Clin Oncol,2010,33(5):474-480.
[17] Jeppesen A N, Jensen H K, Donskov F, et al. Hyponatremia as a prognostic and predictive factor in metastatic renal cell carcinoma[J]. Br J Cancer,2010,102(5):867-872.
[18] Thom I, Andritzky B, Schuch G, et al. Elevated pretreatment serum concentration of YKL-40-An independent prognostic biomarker for poor survival in patients with metastatic nonsmall cell lung cancer[J]. Cancer,2010,116(17):4114-4121.

[19] Akechi T, Okamura H, Okuyama T, et al. Psychosocial factors and survival after diagnosis of inoperable non-small cell lung cancer[J]. Psychooncology,2009,18(1):23-29.
[20] Barbot A C, Mussault P, Ingrand P, et al. Assessing 2-month clinical prognosis in hospitalized patients with advanced solid tumors[J]. J Clin Oncol,2008,26(15):2538-2543.
[21] Umemura S, Segawa Y, Ueoka H, et al. Serum level of arginine-vasopressin influences the prognosis of extensive-disease small-cell lung cancer[J]. J Cancer Res Clin Oncol,2007,133(8):519-524.
[22] Ardizzoni A, Cafferata M A, Tiseo M, et al. Decline in serum carcinoembryonic antigen and cytokeratin 19 fragment during chemotherapy predicts objective response and survival in patients with advanced nonsmall cell lung cancer[J]. Cancer,2006,107(12):2842-2849.
[23] Donskov F, von der Maase H. Impact of immune parameters on long-term survival in metastatic renal cell carcinoma[J]. J Clin Oncol,2006,24(13):1997-2005.
[24] Schmidt H, Johansen J S, Gehl J, et al. Elevated serum level of YKL-40 is an independent prognostic factor for poor survival in patients with metastatic melanoma[J]. Cancer,2006,106(5):1130-1139.
[25] Seve P, Ray-Coquard I, Trillet-Lenoir V, et al. 12 Low serum albumin levels and liver metastasis are powerful prognostic markers for survival in patients with carcinomas of unknown primary site[J]. Cancer,2006,107(11):2698-2705.
[26] Shin H S, Lee H R, Lee D C, et al. Uric acid as a prognostic factor for survival time: a prospective cohort study of terminally ill cancer patients[J]. J Pain Symptom Manage,2006,31(6):493-501.
[27] Berruti A, Mosca A, Tucci M, et al. Independent prognostic role of circulating chromogranin A in prostate cancer patients with hormone-refractory disease[J]. Endocr Relat Cancer,2005,12(1):109-117.
[28] Colinet B, Jacot W, Bertrand D, et al. 06 A new simplified comorbidity score as a prognostic factor in non-small-cell lung cancer patients: description and comparison with the Charlson's index[J]. Br J Cancer,2005,93(10):1098-1105.
[29] George D J, Halabi S, Shepard T F, et al. The prognostic significance of plasma interleukin-6 levels in patients with metastatic hormone-refractory prostate cancer: results from cancer and leukemia group B 9480[J]. Clin Cancer Res,2005,11(5):1815-1820.
[30] Soubrane C, Rixe O, Meric J B, et al. Pretreatment serum interleukin-6 concentration as a prognostic factor of overall survival in metastatic malignant melanoma patients treated with biochemotherapy: a retrospective study[J]. Melanoma Res,2005,15(3):199-204.
[31] Culine S, Kramar A, Saghatchian M, et al. Development and validation of a prognostic model to predict the length of survival in patients with carcinomas of an unknown primary site[J]. J Clin

Oncol,2002,20(24):4679-4683.
[32] Keilholz U, Martus P, Punt C J, et al. Prognostic factors for survival and factors associated with long-term remission in patients with advanced melanoma receiving cytokine-based treatments: second analysis of a randomised EORTC Melanoma Group trial comparing interferon-alpha2a (IFNalpha) and interleukin 2 (IL-2) with or without cisplatin[J]. Eur J Cancer,2002,38(11):1501-1511.
[33] Metintas M, Metintas S, Ucgun I, et al. Prognostic factors in diffuse malignant pleural mesothelioma: effects of pretreatment clinical and laboratory characteristics[J]. Respir Med,2001,95(10):829-835.
[34] Buccheri G, Ferrigno D. 40 Serum biomarkers of non-neuron-endocrine origin in small-cell lung cancer: a 16-year study on carcinoembryonic antigen, tissue polypeptide antigen and lactate dehydrogenase[J]. Lung Cancer,2000,30(1):37-49.
[35] Kudoh K, Kikuchi Y, Kita T, et al. Preoperative determination of several serum tumor markers in patients with primary epithelial ovarian carcinoma[J]. Gynecol Obstet Invest,1999,47(1):52-57.
[36] Ferrari S, Bacci G, Picci P, et al. Long-term follow-up and post-relapse survival in patients with non-metastatic osteosarcoma of the extremity treated with neoadjuvant chemotherapy[J]. Ann Oncol,1997,8(8):765-771.
[37] Rosenfeld M R, Malats N, Schramm L, et al. Serum anti-p53 antibodies and prognosis of patients with small-cell lung cancer[J]. J Natl Cancer Inst,1997,89(5):381-385.
[38] Gray M R, Martin D C S, Zhang X, et al. Metastatic melanoma: lactate dehydrogenase levels and CT imaging findings of tumor devascularization allow accurate prediction of survival in patients treated with bevacizumab[J]. Radiology,2014,270(2):425-434.
[39] Amato R J, Flaherty A, Zhang Y, et al. Clinical prognostic factors associated with outcome in patients with renal cell cancer with prior tyrosine kinase inhibitors or immunotherapy treated with everolimus[J]. Urol Oncol,2014,32(3):345-354.
[40] Cetin B, Afsar B, Deger S M, et al. Association between hemoglobin, calcium, and lactate dehydrogenase variability and mortality among metastatic renal cell carcinoma[J]. Int Urol Nephrol,2014,46(6):1081-1087.
[41] Nakayama T, Saito K, Fujii Y, et al. Pre-operative Risk Stratification for Cancer-specific Survival in Patients with Renal Cell Carcinoma with Venous Involvement Who Underwent Nephrectomy[J]. Jpn J Clin Oncol,2014,44(8):756-761.
[42] Malik L, Parsons H, Mahalingam D, et al. Clinical Outcomes and Survival of Advanced Renal Cancer Patients in Phase I Clinical Trials[J]. Clin Genitourin Cancer,2014.
[43] Faloppi L, Scartozzi M, Bianconi M, et al. The role of LDH serum levels in predicting global outcome in HCC patients treated with sorafenib: implications for clinical management[J]. BMC Cancer,2014,14:110.

[44] Tian Y M, Zeng L, Wang F H, et al. Prognostic factors in nasopharyngeal carcinoma with synchronous liver metastasis: a retrospective study for the management of treatment[J]. Radiat Oncol,2013,8:272.
[45] Almasi C E, Drivsholm L, Pappot H, et al. The liberated domain I of urokinase plasminogen activator receptor--a new tumour marker in small cell lung cancer[J]. APMIS,2013,121(3):189-196.
[46] Khan K, Ang J E, Starling N, et al. Phase I trials in patients with relapsed, advanced upper gastrointestinal carcinomas: experience in a specialist unit[J]. Gastric Cancer,2014.
[47] Bajpai J, Puri A, Shah K, et al. Chemotherapy compliance in patients with osteosarcoma[J]. Pediatr Blood Cancer,2013,60(1):41-44.
[48] Kelly W K, Scher H I, Mazumdar M, et al. Prostate-specific antigen as a measure of disease outcome in metastatic hormone-refractory prostate cancer[J]. J Clin Oncol,1993,11(4):607-615.
[49] Goodman O J, Fink L M, Symanowski J T, et al. Circulating tumor cells in patients with castration-resistant prostate cancer baseline values and correlation with prognostic factors[J]. Cancer Epidemiol Biomarkers Prev,2009,18(6):1904-1913.
[50] Tucci M, Mosca A, Lamanna G, et al. Prognostic significance of disordered calcium metabolism in hormone-refractory prostate cancer patients with metastatic bone disease[J]. Prostate Cancer Prostatic Dis,2009,12(1):94-99.
[51] Sabbatini P, Larson S M, Kremer A, et al. Prognostic significance of extent of disease in bone in patients with androgen-independent prostate cancer[J]. J Clin Oncol,1999,17(3):948-957.

No HR and 95%CI or Pvalues or univariate analysis ： n=26

[1] Berthold F, Kassenbohmer R, Zieschang J. 56 Multivariate evaluation of prognostic factors in localized neuroblastoma[J]. Am J Pediatr Hematol Oncol,1994,16(2):107-115.
[2] Brown J E, Cook R J, Lipton A, et al. 16 Prognostic factors for skeletal complications from metastatic bone disease in breast cancer[J]. Breast Cancer Res Treat,2010,123(3):767-779.
[3] Li Y, Zhang X, Sun Y. [Multivariate analysis of prognostic factors in patients with small cell lung cancer][J]. Zhongguo Fei Ai Za Zhi,2006,9(6):525-529.
[4] Nowecki Z I, Rutkowski P, Kulik J, et al. Molecular and biochemical testing in stage III melanoma: multimarker reverse transcriptase-polymerase chain reaction assay of lymph fluid after lymph node dissection and preoperative serum lactate dehydrogenase level[J]. Br J Dermatol,2008,159(3):597-605.
[5] Rohr U P, Rehfeld N, Pflugfelder L, et al. 23 Expression of the tyrosine kinase c-kit is an independent prognostic factor in patients with small cell lung cancer[J]. Int J Cancer,2004,111(2):259-263.
[6] Sundstrom S, Bremnes R M, Kaasa S, et al. 39 Second-line chemotherapy in recurrent small cell lung cancer. Results from a crossover schedule after primary treatment with cisplatin and etoposide (EP-regimen) or

cyclophosphamide, epirubicin, and vincristin (CEV-regimen)[J]. Lung Cancer,2005,48(2):251-261.
[7] Tas F, Ciftci R, Kilic L, et al. Age is a prognostic factor affecting survival in lung cancer patients[J]. Oncol Lett,2013,6(5):1507-1513.
[8] Marino M T, Grilli A, Baricordi C, et al. Prognostic significance of miR-34a in Ewing sarcoma is associated with cyclin D1 and ki-67 expression[J]. Ann Oncol,2014.
[9] Garrido-Laguna I, Janku F, Vaklavas C, et al. Validation of the Royal Marsden Hospital prognostic score in patients treated in the Phase I Clinical Trials Program at the MD Anderson Cancer Center[J]. Cancer,2012,118(5):1422-1428.
[10] Bajpai J, Puri A, Shah K, et al. Chemotherapy compliance in patients with osteosarcoma[J]. Pediatr Blood Cancer,2013,60(1):41-44.
[11] Formica V, Cereda V, di Bari M G, et al. Peripheral CD45RO, PD-1, and TLR4 expression in metastatic colorectal cancer patients treated with bevacizumab, fluorouracil, and irinotecan (FOLFIRI-B)[J]. Med Oncol,2013,30(4):743.
[12] Bar J, Spencer S, Morgan S, et al. Correlation of lactate dehydrogenase isoenzyme profile with outcome in patients with advanced colorectal cancer treated with chemotherapy and bevacizumab or cediranib: Retrospective analysis of the HORIZON I study[J]. Clin Colorectal Cancer,2014,13(1):46-53.
[13] Nieder C, Marienhagen K, Dalhaug A, et al. Prognostic models predicting survival of patients with brain metastases: integration of lactate dehydrogenase, albumin and extracranial organ involvement[J]. Clin Oncol (R Coll Radiol),2014,26(8):447-452.
[14] Braccini A L, Azria D, Thezenas S, et al. Comparative performances of prognostic indexes for breast cancer patients presenting with brain metastases[J]. BMC Cancer,2013,13:70.
[15] Kamiya N, Suzuki H, Ueda T, et al. Clinical outcomes by relative docetaxel dose and dose intensity as chemotherapy for Japanese patients with castration-resistant prostate cancer: a retrospective multi-institutional collaborative study[J]. Int J Clin Oncol,2014,19(1):157-164.
[16] Tas F, Karabulut S, Ciftci R, et al. Serum levels of LDH, CEA, and CA19-9 have prognostic roles on survival in patients with metastatic pancreatic cancer receiving gemcitabine-based chemotherapy[J]. Cancer Chemother Pharmacol,2014,73(6):1163-1171.
[17] Henry L, Fabre C, Guiraud I, et al. Clinical use of p-proteasome in discriminating metastatic melanoma patients: comparative study with LDH, MIA and S100B protein[J]. Int J Cancer,2013,133(1):142-148.
[18] Margulis V, Shariat S F, Rapoport Y, et al. Development of accurate models for individualized prediction of survival after cytoreductive nephrectomy for metastatic renal cell carcinoma[J]. Eur Urol,2013,63(5):947-952.
[19] Giessen C, Nagel D, Glas M, et al. Evaluation of preoperative serum

markers for individual patient prognosis in stage I-III rectal cancer[J]. Tumour Biol,2014.
[20] Wang H, Zhou N, Xu X, et al. [Surgical treatment of bone metastases from renal cell carcinoma][J]. Zhonghua Yi Xue Za Zhi,2014,94(4):289-292.
[21] Aeckerle S, Moor M, Pilz L R, et al. Characteristics, treatment and prognostic factors of patients with gynaecological malignancies treated in a palliative care unit at a university hospital[J]. Onkologie,2013,36(11):642-648.
[22] Philipp A B, Nagel D, Stieber P, et al. Circulating cell-free methylated DNA and lactate dehydrogenase release in colorectal cancer[J]. BMC Cancer,2014,14:245.
[23] Hou J Y, Aparo S, Ghalib M, et al. Clinical outcome and prognostic markers for patients with gynecologic malignancies in phase 1 clinical trials: a single institution experience from 1999 to 2010[J]. Gynecol Oncol,2013,131(1):163-168.
[24] Soper M S, Hastings J R, Cosmatos H A, et al. Observation Versus Adjuvant Radiation or Chemotherapy in the Management of Stage I Seminoma: Clinical Outcomes and Prognostic Factors for Relapse in a Large US Cohort[J]. Am J Clin Oncol,2014,37(4):356-359.
[25] Sorensen J B, Badsberg J H, Olsen J. Prognostic factors in inoperable adenocarcinoma of the lung: a multivariate regression analysis of 259 patients[J]. Cancer Res,1989,49(20):5748-5754.
[26] Meyers P A, Heller G, Healey J, et al. Chemotherapy for nonmetastatic osteogenic sarcoma: the Memorial Sloan-Kettering experience[J]. J Clin Oncol,1992,10(1):5-15.

Meta-regression Number of obs = 65

REML estimate of between-study variance tau2 = .05335

% residual variation due to heterogeneity I-squared_res = 93.58%

Proportion of between-study variance explained Adj R-squared = -1.72%

With Knapp-Hartung modification

loghr	exp(b)	Std. Err.	t	P>|t|	[95% Conf.	Interval]
cutoff	1.000138	.0002724	0.51	0.614	.999594	1.000683
_cons	1.602006	.1513549	4.99	0.000	1.326385	1.934902
